# A Transnational and Transregional Study of the Impact and Effectiveness of Social Distancing for COVID-19 Mitigation

**DOI:** 10.3390/e23111530

**Published:** 2021-11-18

**Authors:** Tarcísio M. Rocha Filho, Marcelo A. Moret, José F. F. Mendes

**Affiliations:** 1 Instituto de Física and International Center for Condensed Matter Physics, Campus Universitário Darcy Ribeiro, Universidade de Brasília, Asa Norte, Brasília 70919-970, Brazil; 2 Gestão e Tecnologia Industrial (PPG), Centro Universitário SENAI CIMATEC, Salvador 41650-010, Brazil; mamoret@gmail.com; 3 Senai CIMATEC, Universidade do Estado da Bahia—UNEB, Salvador 41150-000, Brazil; 4 Departamento de Física & I3N, Universidade de Aveiro, 3810-193 Aveiro, Portugal; jfmendes@ua.pt

**Keywords:** COVID-19, social isolation, epidemiological model

## Abstract

We present an analysis of the relationship between SARS-CoV-2 infection rates and a social distancing metric from data for all the states and most populous cities in the United States and Brazil, all the 22 European Economic Community countries and the United Kingdom. We discuss why the infection rate, instead of the effective reproduction number or growth rate of cases, is a proper choice to perform this analysis when considering a wide span of time. We obtain a strong Spearman’s rank order correlation between the social distancing metric and the infection rate in each locality. We show that mask mandates increase the values of Spearman’s correlation in the United States, where a mandate was adopted. We also obtain an explicit numerical relation between the infection rate and the social distancing metric defined in the present work.

## 1. Introduction

The current COVID-19 pandemic is the main health crisis in the world in a century, with over 220 million cases and 4.5 million deaths [1]. It began in China at the end of 2019, and has since expanded to every country in the world, with waves occurring at different times in each location. A number of interventions were implemented in most countries, such as travel ban, social distancing and mandatory mask use [2,3], and their effects have been discussed in different works, which generally concluded that they were effective in reducing the growth of cases and deaths [4,5,6,7,8,9]. Possibly, more effective measures are lock-downs, closing of workplaces, businesses school closing, i.e., the social distancing policies [10], with travel restrictions expected to have modest effects in reducing transmission when there is a high circulation of the virus [11].

In order to quantify and qualify the degree of social distancing and its effects, some different approaches have been proposed: by survey questionnaires in the population in order to assess adherence to social distancing and to compare it to the growth of cases, or deaths [12], or by using mobility data from different sources [13,14,15,16,17,18,19]. In the latter case, a mobility or social distancing metric is compared to the growth rate of cases (or deaths) of COVID-19, or to the effective reproduction number $R_{t}$. As we discuss below, this introduces a limitation in the analysis due to the fact that the interpretation of both the growth rate and $R_{t}$ at the beginning of the pandemic, when most of the population is still susceptible to the virus, is different to that at latter stages, when a non-negligible proportion of the population has already been infected, or has already been vaccinated. A more informative parameter, that better represents information on the circulation of the SARS-CoV-2 virus, is the average infection rate $\bar{\beta }$, which is proportional to $R_{t}$ divided by the proportion of the susceptible population (see Equation (3) below). This explains particularly the result by Gatalo et al. [20] who obtained a strong Pearson correlation between phone mobility data and COVID-19 growth rates at earlier stages, but a weaker correlation at later stages, for 25 counties in the United States.

We present here an analysis of the effect of social distancing for 22 European countries and for the 50 and 27 states of the United States and Brazil, and the most populous cities and municipalities for the latter two, respectively. These localities have different situations and histories of the pandemic. For instance, as mask use became mandatory at different moments for American states, we were able to obtain quantitative evidence on its effect on enhancing social distancing policies.

Our main goal is to evidence a monotonous relationship between social distancing data and the value of the infection rate, and to quantify it explicitly.

## 2. Material and Methods

### 2.1. Effective Reproduction Number

The effective reproduction number $R_{t}$ at day *t*, estimated from the generation time distribution w(t) with *t* the number of days between infections, is given by [21]:

(1) $$R_{t}=\frac{I(t)}{\sum_{t^{\prime }}w\left(t^{\prime }\right)I\left(t-t^{\prime }\right)},$$

with I(t) the number (or proportion) of infected individual at day *t*. The effective reproduction number can also be estimated from the series of deaths by first determining the number of infected individuals as:

(2) $$I\left(t\right)=\frac{1}{\bar{\theta }}\sum_{t^{\prime }}N_{\mathrm{deaths}}\left(t+t^{\prime }\right)u\left(t^{\prime }\right),$$

where u(t) is the distribution of the number *t* of days (taken as discrete) between first symptom and death [22], $N_{\mathrm{deaths}}\left(t\right)$ the number of deaths at day *t* and $\bar{\theta }$ is the average infection fatality ratio [23], computed from the demographic structure in each locality. We then use Equation (1) to determine $R_{t}$ at a given day.

### 2.2. Infection Rate

The infection rate can be estimated as [24]:

(3) $$\bar{\beta }=\frac{\gamma R_{t}}{S(t)},$$

with S(t) the proportion of susceptible individuals in the population at day *t*, $R_{t}$ the time-dependent effective reproduction number, and γ the recovery rate from infection with the value reported in the literature [25]. We can also write that

(4) $$\bar{\beta }=P_{c}C,$$

where *C* is the average number of contacts of one individual per day, and $P_{c}$ the probability of contagion of a susceptible individual from a single contact with an infected individual. Social distancing acts by reducing the number of contacts *C*, while other non-pharmaceutical interventions reduce the value of $P_{c}$.

### 2.3. Social Distancing Metric

**Figure 1:**
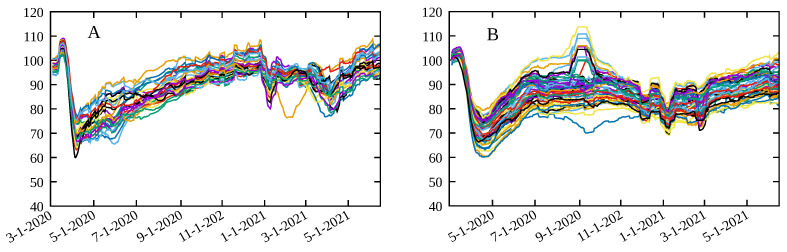
Social distancing metric *M* in Equation (5) for (**A**) Brazil states and (**B**) USA states.

As a proxy for the “amount” of social distancing, we define a metric quantifying the deviation from a baseline representing the pre-pandemic normality. Many possibilities exist, and different mobility data are available from different sources [26,27,28,29]. We require that data are freely available, with coverage up to the city level. For these sources, only Google mobility trends satisfies these two criteria, providing data on the following six categories of locations: retail and recreation ($D_{1}$); grocery and pharmacy ($D_{2}$); parks ($D_{3}$); transit stations ($D_{4}$); workplaces ($D_{5}$) and residential ($D_{6}$), as percentages of variation of time spent in each type of place, with respect to a baseline defined for the period of 3 January to 6 February 2020. The symbols between parenthesis represent the numeric value of the time series for each type of data. An increase in the time spent at residence is expected to decrease the value of the infection rate $\bar{\beta }$, and is considered as a negative contribution to the metric, while an increase in the remaining five categories are expected to increase $\bar{\beta }$ and thus contribute with a positive sign. The social distancing metric is then defined as a weighted average of the data for each category, with the specified sign, with weights given by an (arbitrarily) estimated average proportion of the duration of a day spent in each type of location, and given by

(5) $$M\equiv 100+\frac{0.5}{24}D_{1}+\frac{1}{24}D_{2}+\frac{0.5}{24}D_{3}+\frac{1}{24}D_{4}+\frac{9}{24}D_{5}-\frac{12}{24}D_{6},$$

where the value of 100 is added such that the baseline is close to this value, and has no effect of the value of the Spearman’s correlation. The resulting metric *M* for each Brazilian and American state is shown in Figure 1A,B, respectively, with a similar behavior for the other localities considered here (not shown). This definition is such that a smaller value of *M* represents a more beneficial situation.

### 2.4. Spearman’s Rank-Order Correlation

Spearman’s rank-order correlation $r_{s}\left(A,\;B\right)$ between two time series $A=(A_{1},$ …$,A_{N_{\mathrm{data}}})$ and $B=(B_{1},\ldots ,B_{N_{\mathrm{data}}})$, of length $N_{\mathrm{data}}$, with $A_{i}$ the value of the series at the *i*-th data value, is defined as [30]:

(6) $$r_{s}\left[A,B\right]=1-6\frac{\sum_{i=1}^{N_{\mathrm{data}}}d_{i}^{2}}{N_{\mathrm{data}}\left(N_{d}^{2}-1\right)},$$

such that $-1\geq r_{s}\geq 1$ and $d_{i}$ is the difference in paired ranks of the two series *A* and *B*, i.e., the difference in position of the *i*-th data point for the two datasets when ordered in ascending order. The coefficient $r_{s}$ measures the strength of how two variables are monotonically related, by an increasing or decreasing relation if $r_{s}\;>\;0$ or $r_{s}\;<\;0$, respectively.

**Figure 2:**
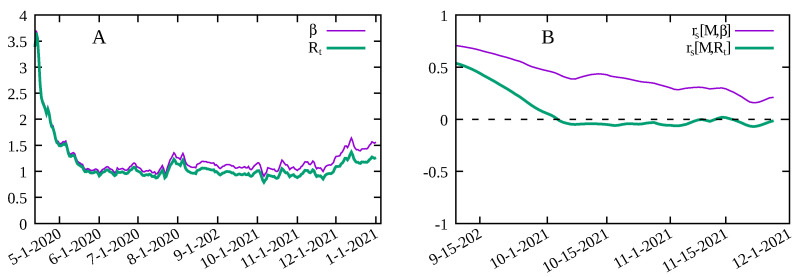
(**A**) Time variation of $\bar{\beta }\left(t\right)$ and $R_{t}$ for Los Angeles county. (**B**) Spearman’s rank-order correlation $r_{s}\left[M,\bar{\beta }\right]$ between the social distancing metric *M* and the infection rate $\bar{\beta }$ and $r_{s}\left[M,R_{t}\right]$ between *M* and the effective reproduction number $R_{t}$ for Los Angeles county in the United States.

In order to show the importance to account for the decreasing number of susceptible individuals with time, we show in Figure 2A the time evolution of $R_{t}$ and $\bar{\beta }$ for the Los Angeles county in the United States. As the proportion of susceptible individuals decreases over time, $R_{t}$ and $\bar{\beta }$ diverge slowly. By computing the Spearman’s correlation between *M* and $R_{t}$ and between *M* and $\bar{\beta }$, for a period of $N_{\mathrm{data}}\;=\;150$ days for the same data, we see from Figure 2B that a small difference between $R_{t}$ and $\bar{\beta }$ has a significant effect on the value of $r_{s}$. The Spearman’s correlation between *M* and $R_{t}$ is close to zero at later times while clearly positive for *M* and $\bar{\beta }$. This is explained by the fact that, from Equation (3), that the same value of $R_{t}$ can correspond to different values of the infection rate $\bar{\beta }$ which is directly related to the circulation of the virus, as it measures the rate at which susceptible individuals are infected, and thus more closely related to the different mitigation policies implemented. We conclude that using $R_{t}$ to represent the stage of the pandemic can lead to misleading results at later stages in assessing the effectiveness of social distancing, as the number of susceptible individuals decreases, and that of vaccinated individuals increase.

### 2.5. Data Sources

The following data sources were employed in the present work:

- Population by age for Europe: World Population Prospects—United Nations—Available online: https://population.un.org/wpp (accessed on 4 September 2021).
- Time series of deaths and cases by country: World Health Organization—Available online: https://www.who.int/emergencies/diseases/novel-coronavirus-2019 (accessed on 4 September 2021).
- Time series of cases and deaths by US counties and states: New York Times COVID-19 Tracker dataset—Available online: https://raw.githubusercontent.com/nytimes/covid-19-data/master/us-counties.csv (accessed on 4 September 2021).
- Population by age group in US counties and states: United States Census Bureau—Available online: https://www.census.gov/data/tables/time-series/demo/popest/2010s-counties-detail.html (accessed on 4 September 2021).
- Data on days of mask mandate in the US: Center for Disease Control and Prevention—Available online: https://data.cdc.gov/Policy-Surveillance/U-S-State-and-Territorial-Public-Mask-Mandates-Fro/62d6-pm5i (accessed on 4 September 2021).
- Population by age group for Brazilian municipalities and states: Brazilian Institute for Geography and Statistics—Available online: https://brasilemsintese.ibge.gov.br/populacao (accessed on 4 September 2021).
- Time series for cases and deaths by COVID-19 by municipality and state in Brazil: Brazilian Ministry of Health—Available online: https://covid.saude.gov.br (accessed on 4 September 2021).
- Detailed data on vaccination in Brazil: Brazilian Ministry of Health—Available online: https://opendatasus.saude.gov.br/dataset/covid-19-vacinacao (accessed on 4 September 2021).

## 3. Results and Discussion

The localities analyzed here are:

- All 50 US states, from the first reported case up to 20 December 2020;
- The 24 US counties with a population of at least one million and at least 1000 deaths in 2020 (Nassau was not considered due to inconsistent data for the number of deaths), from the first reported case in each county up to 20 December 2020;
- All 27 Brazilian states, from 26 February 2020 to 14 June 2021;
- The 22 Brazilian cities (municipalities) with a population of at least 750 thousand from 26 February 2020 to 14 June 2021;
- All countries in the European Economic Community and the United Kingdom with Google Mobility data and at least one thousand deaths by COVID-19 in 2020, from 1 March 2020 to 31 December 2020, with a total of 22 countries.

The span of time of the data was chosen to avoid the effect of vaccination in the United States and Europe, while for Brazil detailed and publicly available anonymized data on each vaccine shot delivered allows modeling the time evolution of the pandemic for a longer period. For estimations of susceptible population in Equation (3), we use the epidemiological model described in [31] to determine the attack rate in each locality and the model is described in Appendix A. Serological surveys also provide such estimates, but are not available for every locality and for the required time window and, where available, data do not have the required time resolution.

**Figure 3:**
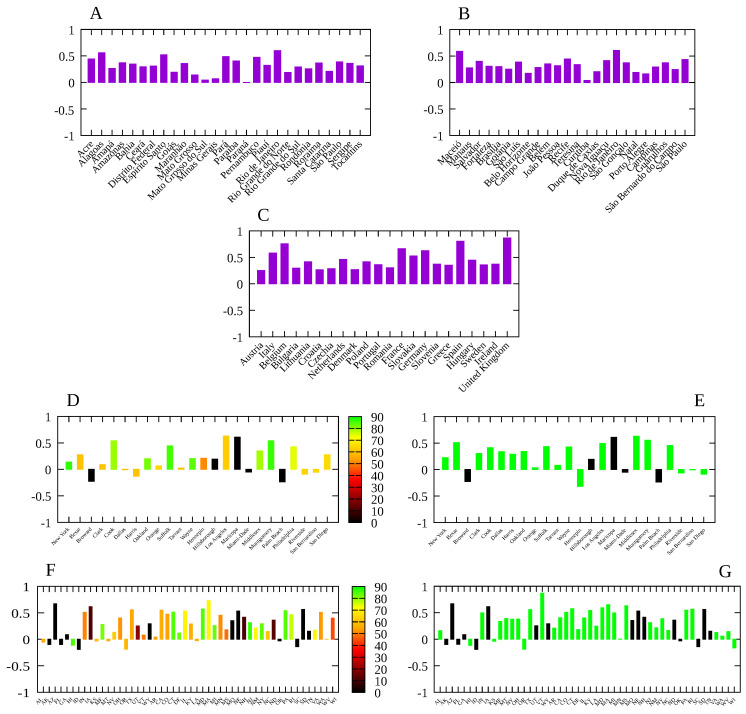
Spearman’s correlation index $r_{s}$ between the social distancing metric *M* and the infection rate $\bar{\beta }$ for: (**A**) Brazilian states; (**B**) Main Brazilian municipalities with population over 750 thousand; (**C**) 22 European countries; (D) US counties with at least one million inhabitants. (**D**) Main counties in the United States. Bar colors give the proportion of days with a mask mandate since the beginning of the pandemic in each location, up to 20 December 2020; (**E**) same as (**D**) but considering only the period with a mask mandate. States without a mask mandate in the period considered are marked in black. (**F**) Same as (**D**) but for all American states. (**G**) Same as (**E**) but for the American states.

**Figure 4:**
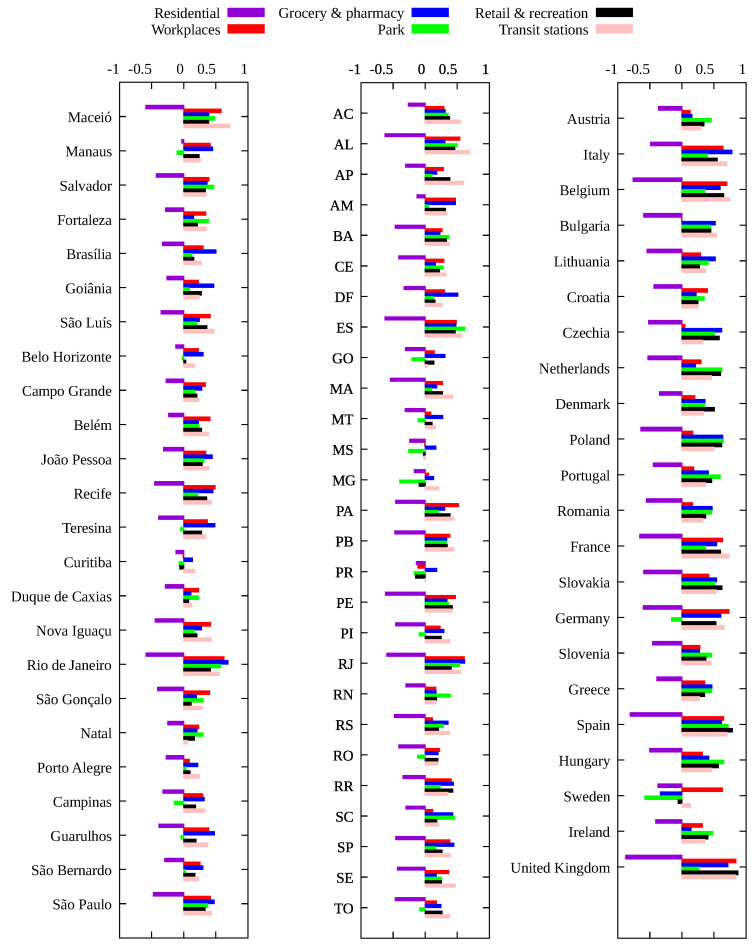
Spearman’s correlation index $r_{s}$ between each mobility variable and the infection rate $\bar{\beta }$ for the main Brazilian municipalities, each Brazilian state and European countries.

**Figure 5:**
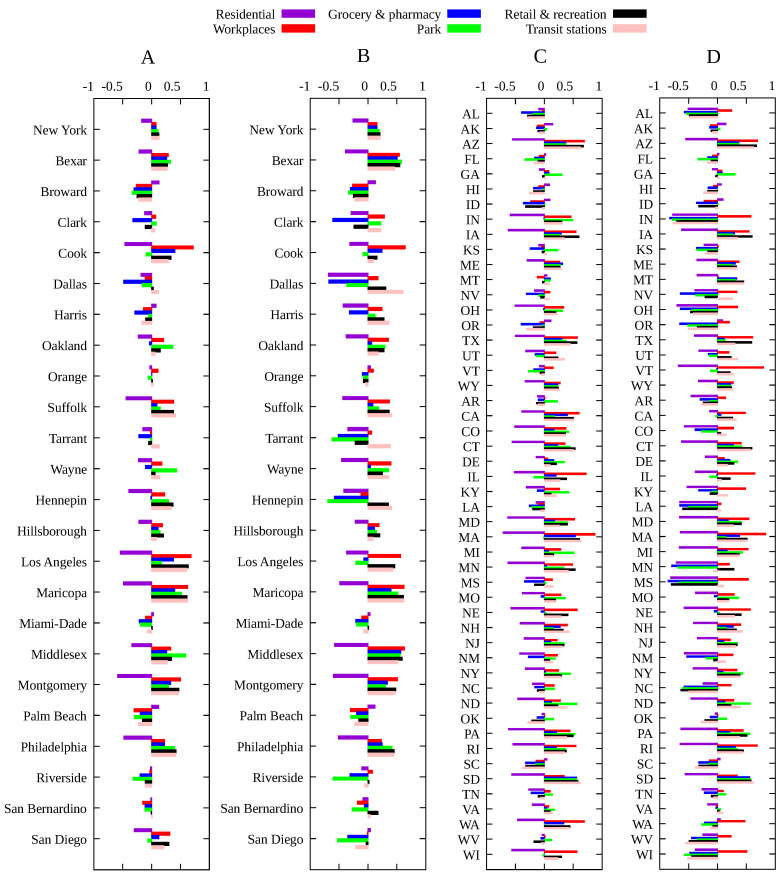
Spearman’s correlation index $r_{s}$ between changes in each mobility category and the infection rate $\bar{\beta }$ for (**A**) counties with more than one million inhabitants and one thousand deaths for the period from the first COVID-19 case up to 20 December 2020; (**B**) same as (**A**) but for the period with a mask mandate, except those counties with no mask mandate in 2020 (marked in black in Figure 3E), for which the whole period is considered; (**C**) same as (**A**) for all US states; (**D**) same as (**B**) for all US states.

**Figure 6:**
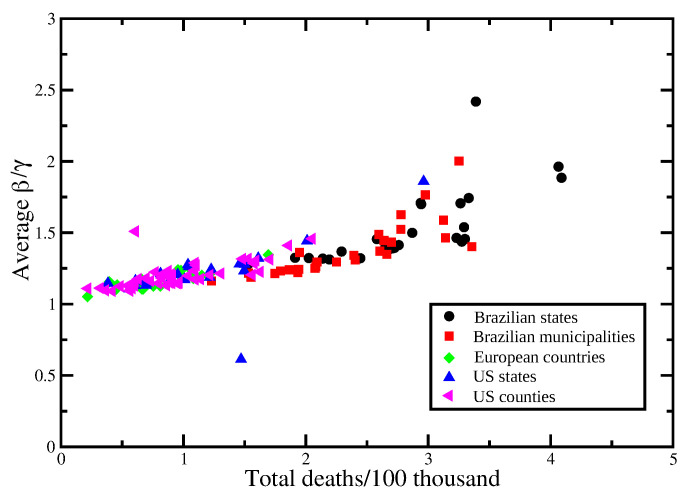
Total number of deaths per 100 thousand inhabitants at the end of the considered period as a function of the average value of $\bar{\beta }/\gamma$ during the same time span.

The results of the Spearman’s rank-order correlation between the social distancing metric *M* and the infection rate $\bar{\beta }$ for each locality are show in Figure 3. In order to assess the effect of mandatory mask use in each US county and state, we compute $r_{s}$ for two periods: for the whole period, indicating in the corresponding graphic the percentage of time with a mask mandate, and for the period with a mask mandate, for those counties with a mandate for at least 50% of the days since the beginning of the pandemic, while for the remaining counties, we consider the whole period and display the corresponding histogram in black. We also computed the Spearman’s correlation separately for each of the six mobility data reported by Google, with results shown in Figure 4 and Figure 5. The average of $\bar{\beta }/\gamma$, over the time period considered for each locality, versus the total number of deaths at the end of each period is shown in Figure 6, where an approximately linear relation is clearly visible, with the exception of a few cases in Brazil.

**Figure 7:**
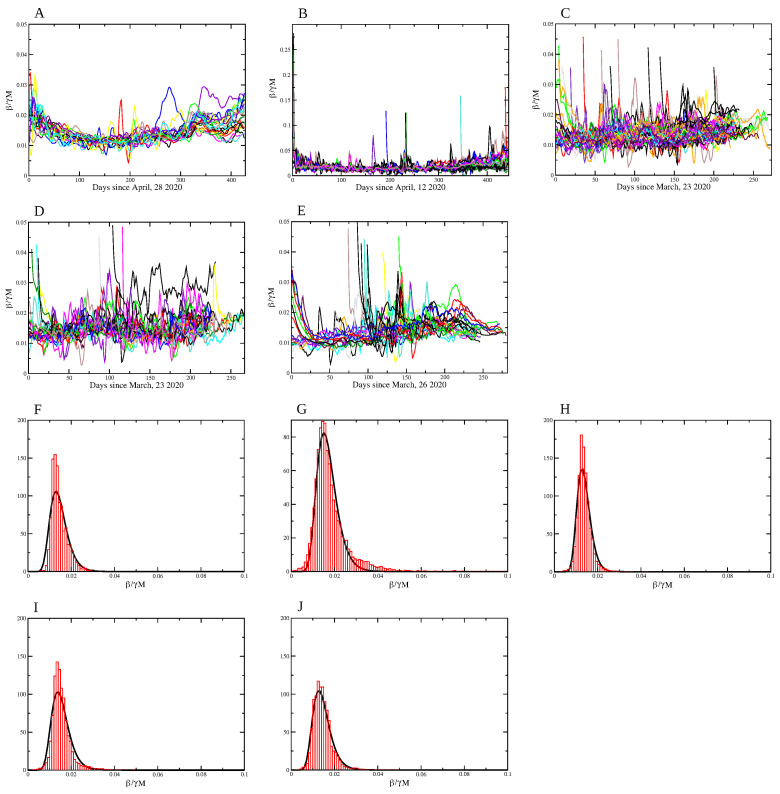
Coefficient $\bar{\beta }/\gamma M=\alpha /\gamma$ for (**A**) Brazilian states; (**B**) Brazilian municipalities; (**C**) US states; (**D**) US counties and (**E**) European countries. The normalized histogram (in red) and the log-normal distribution function (in black) for the values for α/γ: (**F**) Brazilian states; (**G**) Brazilian municipalities, (**H**) US states; (**I**) US counties and (**J**) European countries. The values for α/γ (CI 95%) are 0.015 (0.0096–0.023), 0.019 (0.0081–0.042), 0.014 (0.0089–0.021), 0.015 (0.0091–0.027) and 0.014 (0.0084–0.024), respectively.

In order to established a numeric relationship between $\bar{\beta }$ and *M*, let us assume the linear relation

(7) $$\bar{\beta }\left(t\right)=\alpha M\left(t\right),$$

with α a constant, and consider only the time window that allows to an accurate estimation of $R_{t}$. The distributions of values of the ratio $\alpha /\gamma =\bar{\beta }\left(t\right)/\gamma M\left(t\right)$ for the Brazilian states, Brazilian municipalities, European countries, US states and counties are shown in Figure 7A–E, with values for α/γ (CI 95%) given by 0.015 (0.0096–0.023), 0.019 (0.0081–0.042), 0.014 (0.0089–0.021), 0.015 (0.0091–0.027) and 0.014 (0.0084–0.024), respectively. We also show the best fit with a log-normal distribution for values of α/γ in Figure 7F–J. The fact that the value of α is greater in the Brazilian municipalities may be explained by different factors. It is common in Brazil for people in smaller cities to seek health treatment in the closest biggest city, mainly for a serious condition, and, in this way, deaths by COVID-19 contracted in other localities end up accounted for in the main municipalities, resulting in an increased value of $\bar{\beta }$ and consequently of α/γ also. More crowded places and poorer living conditions in such municipalities may also result in an increase of the transmission rate and, thus, in an increase of α.

While vaccination reduces the proportion of susceptible individuals in the population, it does not alter the relationship of the infection rate $\bar{\beta }$ with social distancing policies with *M* as a proxy, and this was explicitly taken into account in our analysis by using an epidemiological model with vaccination compartments. The approach presented here allowed to evidence a monotonous relationship between the infection rate in each locality and the social distancing metric *M*. It also allowed to explicitly obtain a numeric relationship between $\bar{\beta }$ and a metric for social distancing. Behavioral changes can also have a significant impact on the evolution of any epidemic, and are difficult to include in the current analysis. Nevertheless, the significant values obtained for the Spearman’s correlation indicate the important role that social distancing has played up to now. This is particularly clear in Belgium ($r_{s}=0.75$), Spain ($r_{s}=0.8$) and the United Kingdom ($r_{s}=0.88$), three countries with a high attack rate. The correlation is somewhat smaller for other localities, but nevertheless with significant positive values, clearly indicating an approximately monotonous relationship between the two variables.

For Brazil and the European countries, the results for Spearman’s correlation are quite similar: the variation in time spent at residence is negatively correlated with the infection rate, i.e., the more time spent at home the smaller the value of $\bar{\beta }$, while other categories are positively correlated. For the United States, due to a much greater variety of mitigation policies implemented [13], we see a slightly different picture. In general, time at residence is negatively correlated with $\bar{\beta }$ while time at workplace is positively correlated with the transmission rate, as expected. For the remaining categories (grocery and pharmacy, park, retail and recreation and transit stations), we observe both negative and positive correlations according to the locality, indicating that the most relevant categories are those related to the increase of time spent at home and the decrease of time spent at work places. For the United States case, there is a significant increase in the value of $r_{s}$ when considering only the time period with a mask mandate, which indeed shows its effectiveness.

The values of the proportionality constant α/γ between $\bar{\beta }\left(t\right)/\gamma$ and M(t) are surprisingly close to one another, despite the great differences in the history and implemented policies to mitigate the COVID-19 pandemic. We obtain a log-normal distribution for the value of α/γ (and for α consequently) for all types of localities considered here, with average values significantly close to each other, despite all the differences between countries, implemented mitigation policies, and timings. This points to a universal efficacy of social distancing, enhanced by a mandatory mask use. The explicit linear relation in Equation (7) with the value obtained for the proportionality constant α can be used, for instance, in modeling studies with different scenarios for social isolation.

Of course not only social distancing affects the evolution of the infection rate, causing the variation observed for the Spearman’s correlation for the different localities. We note that even a small increase in $\bar{\beta }$, and thus, a small decrease in *M*, for a long period of time, results in a significant increase in mortality, as can be seen from Figure 6. Our analysis does not grasp the impact of great gatherings of individuals and the possible effect of the so-called superspreading events [20], or the implications of contact tracing.

## 4. Conclusions

A proper choice of a variable to represent the current circulation of the virus is central to assessing the effects of mitigation policies. The infection rate as expressed in Equation (4) is affected by the reduction of social contacts through the average number contacts *C*, and by other implemented protocols, such as mask wearing, that reduce the probability of contagion per contact $P_{c}$. On the other hand, the effective reproduction number $R_{t}$, or any other measure of growth rate of the pandemic, also depends on the current attack rate, and confuses variables in the analysis. The value of $R_{t}$ depends on two factors: the amount of virus in circulation and the proportion of susceptible individuals in the population S(t). For instance, for the same value of $R_{t}=1$ occurring at two different moments of time $t_{1}$ and $t_{2}$ such that $S(t_{1})=1$ (begging of the pandemic) and $S(t_{2})=0.5$ (half of the population already infected) would imply $\bar{\beta }\left(t_{1}\right)=1$ and $\bar{\beta }\left(t_{2}\right)=2$, i.e., the probability of being infected by unit of time at $t=t_{2}$ is the double than for $t=t_{1}$. A smaller infection probability is what is sought by the mitigation measures. We see that the same value of $R_{t}$ can mean different situations depending on the attack rate by the virus, and blurs the analysis when a wider time span is considered such that S(t) varies significantly, as is the case in the data analyzed here. At a given moment of time, social isolation acts on $\bar{\beta }$ but not on S(t). While we expect a monotonous relation between $\bar{\beta }$ and *M*, a monotonous relation between $R_{t}$ and *M* is only evidenced for a shorter time interval such that the proportion of susceptible individuals does no vary significantly. We performed the same analysis for all the localities considered (not shown) by computing the Spearman’s correlation between $R_{t}$ and *M*, and obtained much less significant results, as well exemplified in Figure 2. This is an important point to consider as a more detailed analysis requires a large dataset, and consequently, a significant variation in the proportion of susceptible individuals. Computing Spearman’s correlation, rather than Pearson correlation, for instance, allows us to clearly evidence a monotonous relationship between the social distancing metric as defined here and the infection rate, and computed from the whole time series for each locality.

One limitation of the present work is due to the fact that Spearman’s correlation measures the “amount” of how much one variable is a monotonous function of another variable and that the existence of a time lag for social isolation to affect the evolution of the disease may result in a smaller value for the correlation $r_{s}$. Nevertheless, the approximate (inverse) monotonous relation between social isolation and infection rate is clearly evidenced in our results. We also obtained a strong indication of the positive effect of mask use on controlling the spread of the virus. For localities where a mask mandate was in place, the value of the Spearman’s correlation is usually bigger, as well when considering only the time period with a mask mandate. Further and more detailed studies should be performed to put forward a more direct relation between mask use and the infection rate values.

Future research considering socioeconomic and demographic data would certainly provide valuable information on mitigation strategies targeted at specific groups, such as elders and individuals with comorbidities, as well as the impact of school closure, each considered separately from other factors [32]. We hope that the present work will contribute to a better assessment of the effects of social distancing, and at least partially of mask mandates, on the still ongoing mitigation interventions against the COVID-19 pandemic.

## Data Availability

All data is publicly available in the WEB addresses as states in the text.

## Appendix A. Epidemiological Model

In order to determine the proportion of susceptible individuals in a given locality, we use the approach described in [31] based on the SEIAHRV epidemiological model with variables described in Table A1.

**Table A1:** Variables in the SEIAHRV model reported in [31]. All variables are proportions with respect to the initial population and the index *i* refers to the age-group.

Variable	Description
$S_{i}$	Susceptible individuals
$E_{i}$	Exposed individuals (non-contagious)
$I_{i}$	Infected symptomatic individuals (contagious)
$A_{i}$	Asymptomatic symptomatic individuals (contagious)
$H_{i}$	Hospitalized individuals
$V_{i}^{\left(l,k\right)}$	Vaccinated individual with *l* doses with vaccine of type *k* without primary vaccination failure
$U_{i}^{\left(l,k\right)}$	Vaccinated individual with *l* doses with vaccine of type *k* with primary vaccination failure
$R_{i}$	Recovered individuals.
$d_{l}^{\left(i,k\right)}$	Individuals vaccinated per unit of time with the *l*-th dose of vaccine of type *k*.
$e_{l}^{\left(k\right)}$	Efficacy of vaccine of type *k* withe *l* doses.

The proportion $S_{i}$ of susceptible individuals is obtained from the epidemiological model described in [31], with model equations:

$$\begin{array}{ccc} & & \frac{dS_{i}}{dt}=-\left[\lambda_{i}+\mu^{\prime }+\nu_{1}+\frac{1}{p_{v}}\sum_{k=1}^{K}d_{1}^{\left(i,k\right)}\right]S_{i}\\ & & \;+\kappa^{\prime }\delta_{i,1}+\nu_{i-1}S_{i-1}, \end{array}$$

$$\begin{array}{ccc} & & \frac{dE_{i}}{dt}=\lambda_{i}S_{i}+\lambda_{i}\sum_{k=1}^{K}U_{i}^{\left(k\right)}+\nu_{i-1}E_{i-1}\\ & & \;-\left[\sigma +\mu^{\prime }+\nu_{i}+\frac{1}{p_{v}}\sum_{k=1}^{K}d_{1}^{\left(i,k\right)}e_{1}^{\left(k\right)}\right]R_{i}, \end{array}$$

$$\begin{array}{ccc} & & \frac{dI_{i}}{dt}=\left(1-\chi \right)\sigma E_{i}-\left[\gamma +\mu^{\prime }+\nu_{i}\right]I_{i}+\nu_{i-1}I_{i-1}\\ & & \;-\left(1-\chi \right)\zeta_{i}\sigma E_{i}\left(t-\tau_{1}\right), \end{array}$$

$$\begin{array}{ccc} & & \frac{dA_{i}}{dt}=\chi \sigma E_{i}-\left[\gamma +\mu^{\prime }+\nu_{i}+\frac{1}{p_{v}}\sum_{k=1}^{K}d_{1}^{\left(i,k\right)}e_{1}^{\left(k\right)}\right]A_{i}\\ & & \;+\nu_{i-1}A_{i-1}, \end{array}$$

$$\begin{array}{ccc} & & \frac{dH_{i}}{dt}=-\left[\psi +\mu^{\prime }+\nu_{i}\right]H_{i}+\left(1-\chi \right)\zeta_{i}\sigma E_{i}\left(t-\tau_{1}\right)\\ & & \;-\left(1-\chi \right)\theta_{i}\zeta_{i}\sigma E_{i}\left(t-\tau_{2}\right)+\nu_{i-1}H_{i-1}, \end{array}$$

$$\begin{array}{ccc} & & \frac{dR_{i}}{dt}=\gamma I_{i}+\gamma A_{i}-\left[\mu^{\prime }+\nu_{i}+\frac{1}{p_{v}}\sum_{k=1}^{K}d_{1}^{\left(i,k\right)}e_{1}^{\left(k\right)}\right]R_{i}\\ & & \;+\psi H_{i}+\nu_{i-1}R_{i-1}, \end{array}$$

$$\begin{array}{ccc} & & \frac{dV_{i}^{\left(1,k\right)}}{dt}=-\left[\mu^{\prime }+\nu_{i}\right]V_{i}^{\left(1,k\right)}+\nu_{i-1}V_{i-1}^{\left(1,k\right)}\\ & & \;+e_{1}^{\left(k\right)}d_{1}^{\left(i,k\right)}-e_{1}^{\left(k\right)}d_{2}^{\left(i,k\right)}, \end{array}$$

$$\begin{array}{c} \;\frac{dV_{i}^{\left(2,k\right)}}{dt}=-\left[\mu^{\prime }+\nu_{i}\right]V_{i}^{\left(2,k\right)}+\nu_{i-1}V_{i-1}^{\left(2,k\right)}+e_{2}^{\left(k\right)}d_{2}^{\left(i,k\right)}, \end{array}$$

$$\begin{array}{ccc} & & \frac{dU_{i}^{\left(k\right)}}{dt}=-\left[\lambda_{i}+\mu^{\prime }+\nu_{i}\right]U_{i}^{\left(k\right)}+\nu_{i-1}U_{i-1}^{\left(k\right)}\\ & & \;+\frac{\left(1-e_{1}^{\left(k\right)}\right)d_{1}^{\left(i,k\right)}S_{i}}{p_{v}}-\left(e_{2}^{\left(k\right)}-e_{1}^{\left(k\right)}\right)d_{2}^{\left(i,k\right)}, \end{array}$$

(A1) $$\begin{array}{ccc} & & \;p_{v}=S_{i}+E_{i}+A_{i}+R_{i}. \end{array}$$

This is a nonlinear delayed set of ODEs due to the time delay between infection, hospitalization and death. The different parameter values used in the model are given in Table 1 in [31]. The force of infection in Equation (A1) is given by

(A2) $$\lambda_{i}=\sum_{j=1}^{M}\beta_{i,j}\frac{I_{j}+\xi A_{j}}{n_{i}},$$

with $\beta_{i,j}$ the infection rate from an infected individual of age group *j* to infect an individual of age-group *i*. The epidemiological model is calibrated using the time series of deaths in order to avoid the significant under-notification of cases [33]. The value $\bar{\beta }$ in Equation (3) is an age-independent estimate obtained from the total proportion of susceptible individuals obtained from

(A3) $$S\left(t\right)=\frac{1}{P_{\mathrm{tot}}}\sum_{i}P_{i}S_{i}\left(t\right),$$

where $P_{i}$ is the population in age group *i* and $P_{\mathrm{tot}}$ the total population for the given locality. The model is fitted from the time series of deaths as described in [31].
